# Characterization of Peroxidase and Laccase Gene Families and In Silico Identification of Potential Genes Involved in Upstream Steps of Lignan Formation in Sesame

**DOI:** 10.3390/life12081200

**Published:** 2022-08-08

**Authors:** Yedomon Ange Bovys Zoclanclounon, Michael Rostás, Nam-Jin Chung, Youngjun Mo, Petr Karlovsky, Komivi Dossa

**Affiliations:** 1 Genomics Division, National Institute of Agricultural Sciences, Rural Development Administration, Jeonju 54874, Korea; 2 Department of Crop Science and Biotechnology, Jeonbuk National University, Jeonju 54896, Korea; 3 Molecular Phytopathology and Mycotoxin Research, Grisebachstrasse 6, Georg-August-University Goettingen, D-37077 Goettingen, Germany

**Keywords:** specialized metabolites, lignan biosynthesis, sesamin, transcriptomics, oxidative rearrangement

## Abstract

Peroxidases and laccases are oxidative enzymes involved in physiological processes in plants, covering responses to biotic and abiotic stress as well as biosynthesis of health-promoting specialized metabolites. Although they are thought to be involved in the biosynthesis of (+)-pinoresinol, a comprehensive investigation of this class of enzymes has not yet been conducted in the emerging oil crop sesame and no information is available regarding the potential (+)-pinoresinol synthase genes in this crop. In the present study, we conducted a pan-genome-wide identification of peroxidase and laccase genes coupled with transcriptome profiling of diverse sesame varieties. A total of 83 and 48 genes have been identified as coding for sesame peroxidase and laccase genes, respectively. Based on their protein domain and *Arabidopsis thaliana* genes used as baits, the genes were classified into nine and seven groups of peroxidase and laccase genes, respectively. The expression of the genes was evaluated using dynamic transcriptome sequencing data from six sesame varieties, including one elite cultivar, white vs black seed varieties, and high vs low oil content varieties. Two peroxidase genes (*SiPOD52* and *SiPOD63*) and two laccase genes (*SiLAC1* and *SiLAC39*), well conserved within the sesame pan-genome and exhibiting consistent expression patterns within sesame varieties matching the kinetic of (+)-pinoresinol accumulation in seeds, were identified as potential (+)-pinoresinol synthase genes. Cis-acting elements of the candidate genes revealed their potential involvement in development, hormonal signaling, and response to light and other abiotic triggers. Transcription factor enrichment analysis of promoter regions showed the predominance of *MYB* binding sequences. The findings from this study pave the way for lignans-oriented engineering of sesame with wide potential applications in food, health and medicinal domains.

## 1. Introduction

Sesame (*Sesamum indicum* L.), a member of Pedaliaceae family, is an oil crop whose seeds contain lignans including (+)- sesamin, (+)- sesamolin, and (+)- sesaminol [1,2]. Therapeutical properties of sesame lignans against neurodegenerative diseases [3,4], prostate, and breast cancers [5] have been reported. Besides, lignans represent an emerging perspective for health care and disease prevention as functional foods and nutraceuticals [6,7,8,9,10,11,12]. The lignan market is exploding and may reach over USD 610 Million by 2028 [13] with multiple applications covering food, pharmaceutics, and cosmetics industries. Meanwhile, several patents have been deposited concerning the extraction, purification, and transformation of lignans from sesame [14,15,16,17,18,19,20,21,22], showing the growing interest in this class of plant-specialized metabolites.

**Figure 1:**
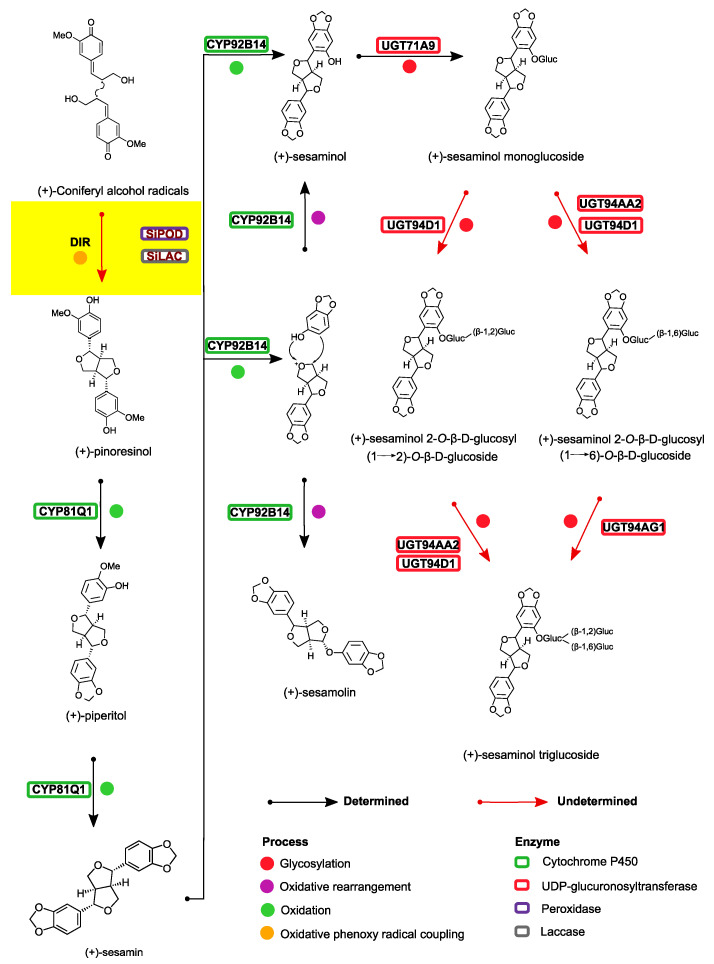
A simplified lignan biosynthesis pathway in sesame showing the route of synthesis of (+)- sesamin, (+)- sesamolin, and (+)- sesaminol. *CYP81Q1* and *CYP92B14* triggered the biosynthesis of (+)- sesamin, (+)- sesamolin and (+)- sesaminol, while *UTG71A9*, *UGT94D1*, *UGT94AA2*, and *UGT94AG1* are suggested to catalyze the synthesis of (+)- sesaminol monoglucoside, (+)-sesaminol 2-O-β-D-glucosyl (1 2)-O-β-D-glucoside, (+)-sesaminol 2-O-β-D-glucosyl (1 6)-*O*-β-D-glucoside, and (+)-sesaminol triglucoside. The target step in the present study is marked by a yellow rectangle. The pathway is adapted from Ono et al. [23].

To date, tremendous works have been done to elucidate the lignan biosynthesis pathway in sesame by using wild (*S. alatum* and *S. radiatum*) and cultivated (*S. indicum*) material [1,23,24,25,26]. A total of six enzymes involved in lignan biosynthesis in sesame have been characterized, including two cytochrome P450 coding genes (*CYP81Q1* and *CYP92B14*), and four glycosyltransferases (*UGT71A9*, *UGT94D1*, *UGT94AG1*, and *UGT94AA2*) [1,25]. The connections between the identified enzymes and their respective targets are depicted in Figure 1. At the initial step of the lignan biosynthesis pathway, an oxidative coupling reaction involving two molecules of conyferol alcohol takes place. With the help of a dirigent protein (*DIR*), the primary precursor of lignan, (+)-pinoresinol, is generated. The latter is then sequentially converted by *CYP81Q1* to produce (+)-piperitol and (+)-sesamin. Further, (+)-sesamolin and (+)-sesaminol synthesis is guided by *CYP92B14*.

It is worth mentioning that the mechanism for the synthesis of the central precursor *ie* (+)-pinoresinol, very likely involves a combinatory action of oxidase enzymes (laccases and/or peroxidases) with a dirigent protein [27]. Oxidation by peroxidases and/or laccases followed by a stereo-selective radical coupling guided by a dirigent protein is assumed to take place at an early stage of the synthesis of all plant lignans [27,28,29,30]. In brief, one-electron oxidation on conyferyl alcohol is catalyzed by peroxides and/or laccasses. As a result, an intermediate molecule, a conyferyl alcohol (1)-derived free radical, is formed. Then an intramolecular cyclization guided by a dirigent protein induces the formation of (+)-pinoresinol molecule [27].

To the best of our knowledge, evidence of oxidase activity involved in the critical precursor step of sesame lignan biosynthesis has not been yet established. While a dirigent protein (*XP_011080883*) has been detected [2] in sesame by sequence homology, the proof of its function as well as the identity of the peroxidase or laccase responsible for the conversion of coniferyl alcohol to (+)-pinoresinol remain to be established. 

Peroxidases and laccases are multifunctional enzymes playing a wide range of roles in plants covering biotic [31,32,33] and abiotic responses [34,35,36,37,38], and other biological processes such as fiber initiation [39], cell elongation [40], lignification [41,42], seed setting and panicle branching [43], pigmentation [44,45], and flavonoid oxidation [46]. Peroxidases and laccases are also used in therapeutic and industrial applications. For instance, peroxidases are used in industrial chemical synthesis, diagnostic tests, and enzyme immunoassays [47]. Meanwhile, laccase has great importance in the paper industry due to its capacity for delignification [48,49]. Moreover, laccases are also useful in ethanol production, wine clarification, industrial effluents treatment, herbicide degradation, dyes decoloration, and drug analysis [50].

Owing to the importance of the peroxidases and laccases in plants, the present study was carried out to comprehensively characterize these enzymes in sesame and outline candidate genes likely involved in the early steps of lignan biosynthesis. 

## 2. Materials and Methods

### 2.1. Genome-Wide Identification of Peroxidase and Laccase Genes and Core Genes Inference

Putative peroxidase and laccase genes were searched for in the genome data of *S. indicum* var. Zhongzhi13 (NCBI RefSeq accession: GCF_000512975.1), *S. indicum* var. Goenbaek (https://zenodo.org/record/6350881, accessed on 15 March 2022), *S. indicum* cv Mishouzhima, *S. indicum* cv. Baizhima, *S. indicum* var. Yuzhi11, and *S. indicum* var. Swetha (http://www.sesame-bioinfo.org/pan-genome, accessed on 15 November 2018).

NCBI HMM accession TIGR03390.1 (EC 1.10.3.2) served for the identification of candidate laccase genes, whereas PFAM HMM accession PF00141.26 was employed to detect peroxidase genes. After a hit search using hmmsearch (-E 1 × 10^−5^—domE 1 × 10^−5^), a domain verification was executed with PfamScan v1.6 [51] to check the presence of the POD (peroxidase) and LAC (laccase) domains. Spurious genes were filtered out. An additional check of the presence of the POD and LAC domains was performed using the InterProScan v5 [52]. 

In order to infer the core conserved POD and LAC genes within the sesame pangenome dataset, Orthofinder v2.3.12 [53] was run with the default settings. 

The identified POD and LAC genes from the reference genome of *S. indicum* var Zhongzhi13 were retained for downstream analysis.

### 2.2. Chromosome Location and Syntheny Analyses

Genome mapping of *SiPOD* (peroxidase genes) and *SiLAC* (laccase genes) was rendered using MG2C V2.1 [54] based on the annotation information. Sesame-to-sesame and sesame-to-Arabidopsis synteny blocks were investigated with the MCScanX toolkit [55]. Regarding the evolutionary origin of the duplicated genes, the duplicate genes classifier Perl script from MCScanX helped to distinguish between genes evolved by tandem or segmental duplication.

### 2.3. Phylogenetic Analysis

Peroxidase and laccase genes from *A. thaliana* were added to *SiPOD* and *SiLAC* genes for the construction of phylogenetic trees. Prior to the tree inference, the genes were aligned using MAFFT v7.464-0 [56]. The resulting alignment was trimmed with trimAl v1.4.1 [57]. Subsequently, the trees were constructed with IQ-TREE v1.6.12 [58]. Peroxidase and laccase maximum likelihood trees were inferred following models LG+R5 and LG+I+G4 with 1000 iterations, respectively. The tree models were selected using the ModelFinder package [59].

### 2.4. RNA-Seq Data Retrieval

A set of six sesame varieties (Zhongzhi13, Zhongfengzhi No.1, Zhongzhi No.33, ZZM4728, ZZM2161, and ZZM3495) were selected for the investigation of peroxidase and laccase gene expression using RNA sequencing data (Table S1). The criteria for the selection were (a) oil content (high oil-producing variety versus low oil-producing variety) and (b) seed color (white versus black). Thus, in addition to the multi-organs transcriptome data of the reference genome Zhongzhi13 [60], the seed RNA-Seq of two pure lines of sesame Zhongfengzhi No.1 and Zhongzhi No.33 [61], exhibiting white and black seed color respectively, were used. The seed transcriptome sequences of one high (59.1%) oil content (ZZM4728) and two low oil content ZZM2161 (48.4%) and ZZM3495 (51.0%) varieties [62], were also downloaded from NCBI. The sesame varieties were grown under identical growth conditions at the Oil Crops Research Institute (OCRI) experimental station in Hubei Province, Wuhan, China, at N 30.57°, E 114.30°, altitude 27 m [60,61,62]. The RNA samplings were performed at 10, 20, 25, and 30 days after anthesis for ZZM2161, ZZM3495, and ZZM4728, while Zhongfengzhi No.1 and Zhongzhi No.33 were sampled at 5, 8, 11, 14, 17, 20, 23, 26, and 30 days after anthesis. Detailed information regarding the materials and RNA-Seq raw data SRAs are provided in Table S1.

### 2.5. Expression Profile Analysis and Candidate Genes Selection

The RNA-Seq raw data were quality-checked using FastQC v0.11.2 [63]. Sequencing adapters and low-quality (Q < 30) reads were filtered out with Trimmomatic v0.36 [64]. The clean data were then mapped to the reference genome using HISAT v2.2.1 [65]. The gene expression profile in each tissue was assessed with the RSEM package v1.3.3 [66] as fragments per kilobase of transcript per million fragments mapped (FPKM). Heatmaps showing the expression in different tissues were plotted with TBTools v1.098746 [67] with log2(FPKM) values.

To select candidate genes, we applied three major filtering criteria. Firstly, from the reference genome dataset, the genes of interest should be preferentially expressed in the seed tissue compared to the root, leaf, stem, and capsule. Secondly, the candidate genes from the first filtering step have been checked for their expression in high versus low oil content varieties with a particular emphasis on those that were expressed early during seed development. The early-stage criterion was included since pinoresinol is the precursor of all lignans in sesame (Figure 1). Additionally, the kinetic of the biosynthesis of the pinoresinol described by Ono et al. [23] implied an increasing expression at the early stage of the seed development that enables later accumulation of downstream lignans in the seed at the maturity stage. Thirdly, after the second filtering step, the same approach was applied to the white versus black seed dataset. It is worth mentioning that the seed color was considered in this study because it has been reported that white sesame seeds contain higher levels of lignans (sesamolin and sesamin) compared to black sesame seeds [68,69]. 

Overall, the retained candidate genes belonged to the core gene repertoire from the sesame pan-genome; were preferentially expressed in seed tissues; and were strongly expressed at the early stage of the seed development.

### 2.6. Conserved Motifs, Gene Structure, GO Annotation, and Orthologs Detection of Candidate SiPOD and SiLAC Genes

The candidate genes from the three filtering steps were screened for conserved motifs using MEME Suite v5.0.4 [70] with a maximum number of motifs set to 20. A 2 Kbp promoter sequence of candidate genes were submitted to the PlantCARE database (http://bioinformatics.psb.ugent.be/webtools/plantcare/html/, accessed on 6 February 2022) [71] to find out cis-acting regulatory elements. Besides, the InterProScan v5 [52]) was employed to find out molecular functions associated with the candidate genes. Meanwhile, their orthologs search was executed with SHOOT (https://www.shoot.bio/, accessed on 6 February 2022) [72] using the plant database option. 

### 2.7. Transcription Factor Enrichment Analysis

Taking advantage of the Plant Transcriptional Regulatory Map (PlantRegMap) platform [73], we performed a transcription factor (TF) enrichment analysis (http://plantregmap.gao-lab.org/tf_enrichment.php, accessed on 8 February 2022) in order to estimate the most contributive TF families potentially involved in the regulation of peroxidase and laccase genes.

### 2.8. Gene Expression Assays Using qRT-PCR

The expression profile of the candidate genes was verified by performing a qRT-PCR in a LightCycler^®^ 480II real-time PCR instrument (Roche Diagnostics, Rotkreuz, Switzerland). Prior to the PCR, five pairs of primers (Table S2) were designed using the primer 3 blast online tool (https://www.ncbi.nlm.nih.gov/tools/primer-blast/, accessed on 11 April 2022). The qRT-PCR experiment was carried out according to Dossa et al. [74]. A total of three independent replicates were applied for each gene. The sesame actin gene (NCBI gene ID: LOC105159390) served as a positive control. The 2^−ΔΔCT^ method [75] was employed to calculate the relative expression of the target genes.

## 3. Results and Discussion

### 3.1. Variability of Peroxidase and Laccase Genes in Sesame Pangenome and Phylogenetic Analyses

**Figure 2:**
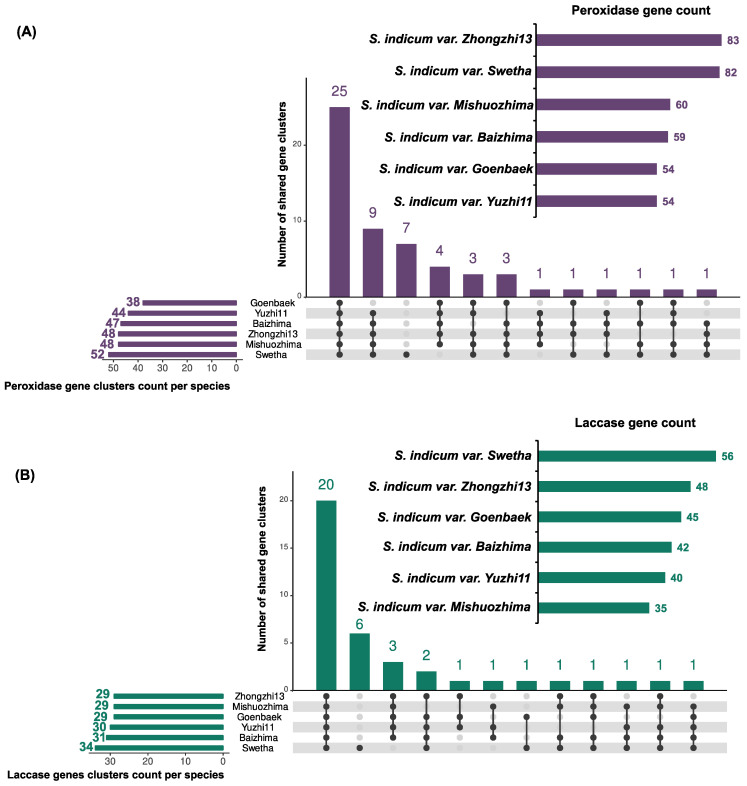
Gene count and conservation of peroxidase (**A**) and laccase (**B**) genes in sesame pangenome. Horizontal bar charts summarize the gene count. Upset plots show the core conserved count of peroxidases/laccases within sesame pan-genome.

From the sesame pan-genome gene sets, 83, 82, 60, 59, 54, and 54 peroxidase genes were counted in Zongzhi13, Swetha, Mishuozima, Baizhima, Goenbaek, and Yuzhi11 genomes, respectively (Figure 2). Similarly to the peroxidase count variability observed at the intra-species level in sesame, high inter-species variability was observed with 138, 119, 102, 90, 73, and 47 peroxidase genes counted in *Oryza sativa* [76], *Zea mays* [77], *Solanum tuberosum* [78], *Betula pendula* [79], *A. thaliana* [80], and *Vitis vinifera* [81], respectively. Most of the gene clusters (25) were shared by all varieties while only Swetha exhibited species-specific gene clusters (7).

An average of 44 ± 7 laccase genes were identified in the sesame pangenome. The most abundant laccases were observed in Swetha (n = 56), followed by Zhongzhi13 (n = 48), Goenbaek (n = 45), Baizhima (n = 42), Yuzhi11 (n = 40), and Mishuozhima (n = 35). Similar laccases counts were observed in land plants *Prunus persica* (n = 48) [82], *Panicum virgatum* (n = 49) [83], and *Solanum melongena* (n = 42) [84]; a lower number of laccase genes was reported in *A. thaliana* (n = 17) [85].

The orthology analysis revealed that the core laccase genes were grouped into 20 clusters, while Swetha harbored 6 specific gene clusters. Except for Yuzhi11 and Goenbaek, the peroxidase and laccase genes were globally abundant in modern varieties (Zhongzhi13, Swetha) compared to landraces (Mishuozima and Baizhima); suggesting the influence of the human oil-oriented selection. In fact, at a whole-genome scale, landraces (Mishuozima and Baizhima) exhibited specific genes coding for environmental adaption, while modern varieties showed preferential genes with oil-related functional attributes [86].

Interestingly, only Swetha showed a specific gene cluster suggesting a unique peroxidase and laccase gene repertoire in this variety. However, this should be interpreted with caution since the Swetha chromosome-scale genome was constructed based on the reference genome of Zhongzhi13 [86] with short-reads assembly as an initial contigs-level assembly.

For downstream analyses, peroxidases and laccases genes from the reference genome Zhongzhi13 were used (Tables S3 and S4).

**Figure 3:**
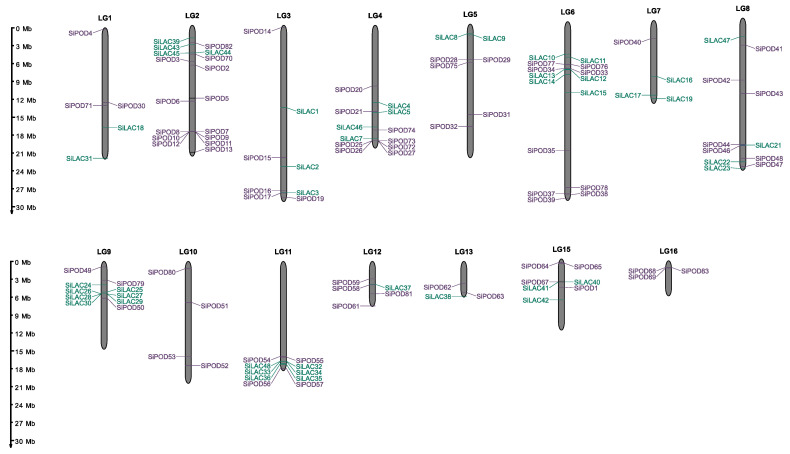
Chromosome location of peroxidase and laccase genes in sesame.

After locating the identified genes on the chromosomes (Figure 3), we assessed the evolutionary determinants of the distribution of *SiPOD* and *SiLAC* genes within the sesame genome. Two types of gene relationships were found (Figures S1 and S2): paralogous genes that are adjacent on the same chromosome (tandem-duplicated genes) and genes that are far away from each other, often located on different chromosomes (segmental duplicated genes). For *SiPOD*, the results highlighted 18 pairs paralogous genes that underwent segmental duplication, while one pair originated from tandem duplication (Figure S1). Tandem and segmental duplications are considered as an evolutionary driving force resulting in gene families’ expansion [87,88]. Peroxidase genes duplication via tandem and segmental duplications were extensively reported in plants, including soybean [36], cassava [89], carrot [90], potato [78], cotton [91], watermelon [92], maize [77], tomato [93], Chinese pear [94] and others. In our study, peroxidase gene expansion mainly resulted from segmental duplication, which is consistent with the findings of Cao et al. [94] in pear. However, in trees such as *Betula pendula* [79] and *Populus trichocarpa* [95], tandem duplication was the main driver of peroxidase gene expansion.

Similarly, a total of 20 *SiLAC* genes belonged exclusively to the segmental duplication group (Figure S2). In contrast, only a tandem duplication pattern was detected in *Solanum melongena* with 16 laccase genes [84]. In *Panicum virgatum*, both tandem and segmental duplications of laccase genes were found [83].

The syntheny analysis revealed that 31 (37%) *SiPOD* and 26 (54%) *SiLAC* genes showed syntheny with *A. thalina* respective gene sets (Figures S3 and S4), suggesting that they are conserved within these species.

**Figure 4:**
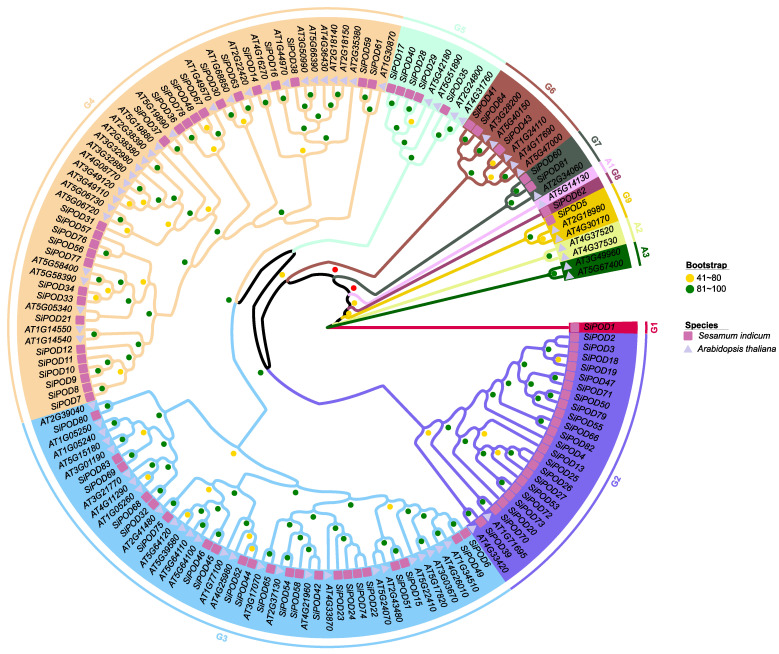
Unrooted maximum likelihood phylogenetic tree inferred from 73 *A. thaliana* and 83 *S. indicum* peroxidase genes. The tree was constructed following the LG+R5 model with a total of 1000 iterations. Square symbol represents genes from *S. indicum* while triangle symbol stands for *A. thaliana* genes. Yellow (41–80), and green (81–100) dot represents the clades support values. Arabidopsis-specific phylogenetic groups were designated A1 to A3. Groups containing *SiPOD* are designated G1 to G9.

**Figure 5:**
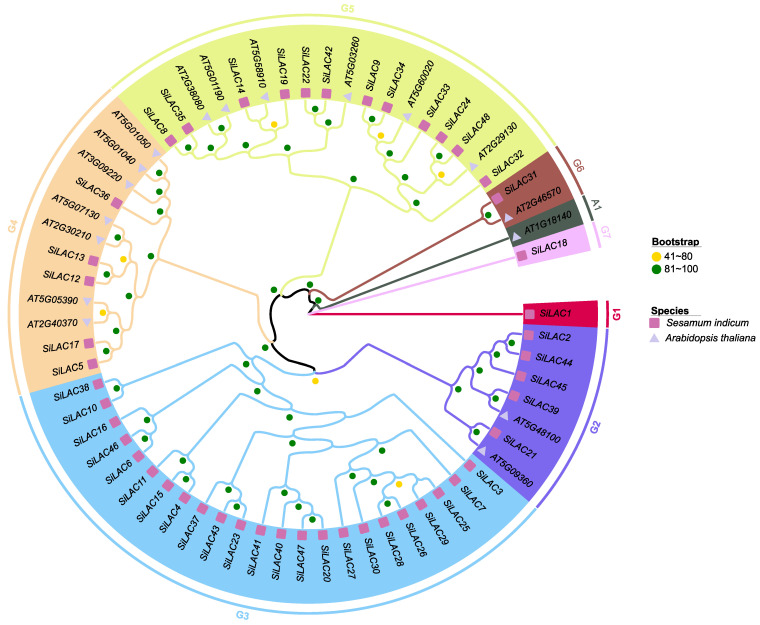
Unrooted maximum likelihood phylogenetic tree inferred from 17 *A. thaliana* and 48 *S. indicum* laccase genes. The tree was constructed following the LG+I+G4 model with a total of 1000 iterations. Square shape represents the genes from *S. indicum* while triangle shape stands for *A. thaliana* genes. Yellow (41–80), and green (81–100) dot represents the clades support values. An Arabidopsis-specific phylogenetic group was designated A1. Groups containing *SiLAC* are designated G1 to G7.

To classify the identified peroxidase and laccase genes, phylogenetic trees were constructed using *A. thaliana* genes as baits. The results revealed nine and seven groups for peroxidase and laccase gene families, respectively (Figure 4 and Figure 5). The tree topology was in line with sesame-alone peroxidase and laccase genes trees (Figures S5 and S6).

### 3.2. Expression Profiles of SiPOD and SiLAC Genes in Different Tissues and Candidate Genes Selection

To identify potential (+)-pinoresinol synthase genes, a multi-varieties comparative transcriptome approach was utilized. RNA-Seq data from six sesame varieties (Table S1) were inspected following the three filtering steps including (i) preferential expression in seed, (ii) expression at least at the early stage of the seed development, and (ii) expression of the gene across all tested varieties. 

**Figure 6:**
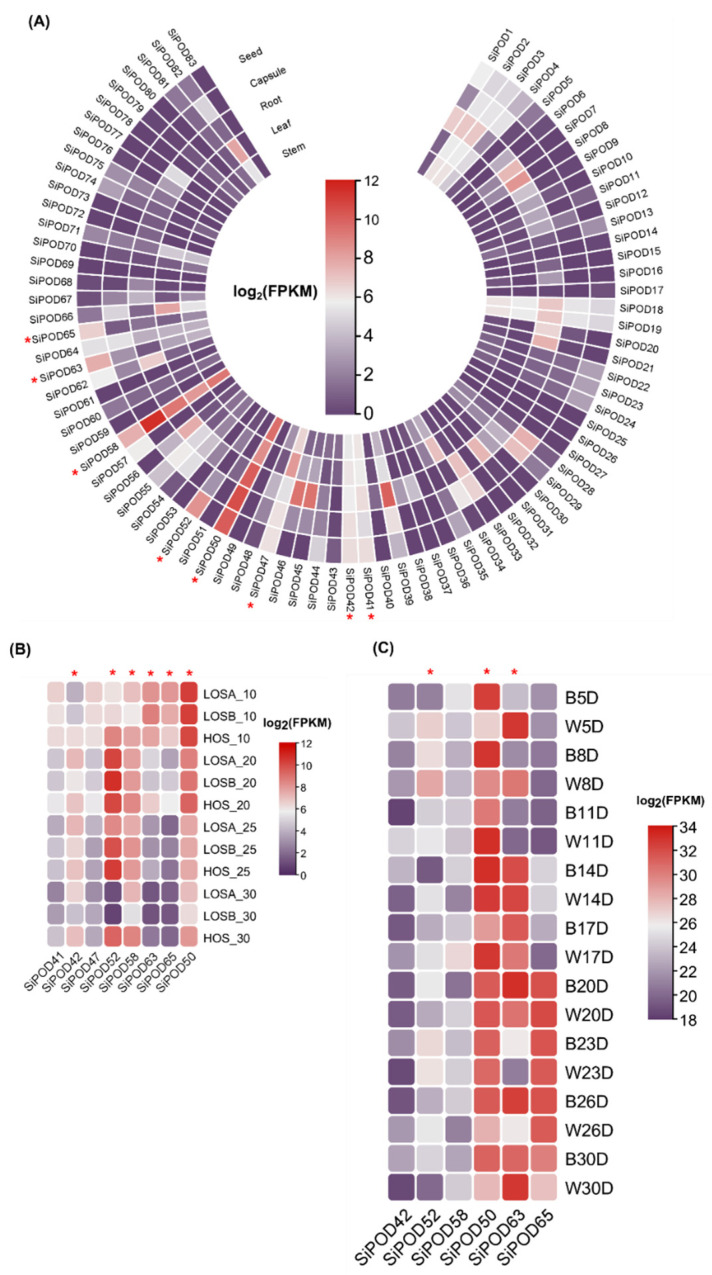
Expression profile of sesame peroxidase genes in different tissues: (**A**) seed, root, leaf, stem and capsule from Zhongzhi13; (**B**) Seed from ZZM4728 (HOS), ZZM3495 (LOSA), and ZZM2161 (LOSB); (**C**) Seed from black seed Zhongzhi No. 33 (B) and white seed Zhongzhi No.1 (W) varieties. Candidate genes for each set of tissues are highlighted with a red star symbol.

For peroxidase genes (Figure 6), a set of eight SiPOD (*SiPOD41*, *SiPOD42*, *SiPOD47*, *SiPOD50*, *SiPOD52*, *SiPOD8*, *SiPOD63*, and *SiPOD65*) genes were preferentially expressed in the seeds of Zhongzhi 13 (Figure 6A).

The selected genes were mined for their expression in the seeds of low (LOS) versus high (HOS) oil content varieties (Figure 6B). The results pinpointed *SiPOD42*, *SiPOD52*, *SiPOD58*, *SiPOD63*, *SiPOD65*, and *SiPOD50*. *SiPOD42* was most expressed in HOS at 10 days as compared to the two LOS varieties. Its expression was maintained within all the seed development stages preferentially in the HOS. Similar observations were noted for *SiPOD52*, *SiPOD58*, and *SiPOD50* with an ascending expression from 10 to 20 days after anthesis followed by a decline starting from 25 days after anthesis. Interestingly, *SiPOD63* and *SiPOD65* were expressed at the early stage (10 days after anthesis) in all varieties before the expression fell in the following development stages. 

The candidate genes from the second filtering round were screened for preferential high expression in seed or at least expression at an earlier stage of seed development regardless of seed coat color (Figure 6C). Thus, the genes *SiPOD52*, *SiPOD63*, *SiPOD50*, and *SiPOD65* came up to be the potential candidates. *SiPOD52* and *SiPOD63* showed a higher expression in white seed at early stage (five and eight days after anthesis). Intriguingly, a higher expression of *SiPOD50* was noted in black seed at five and eight days after anthesis. However, the expression of the gene was quite stable within all development stages, regardless of the seed color.

**Figure 7:**
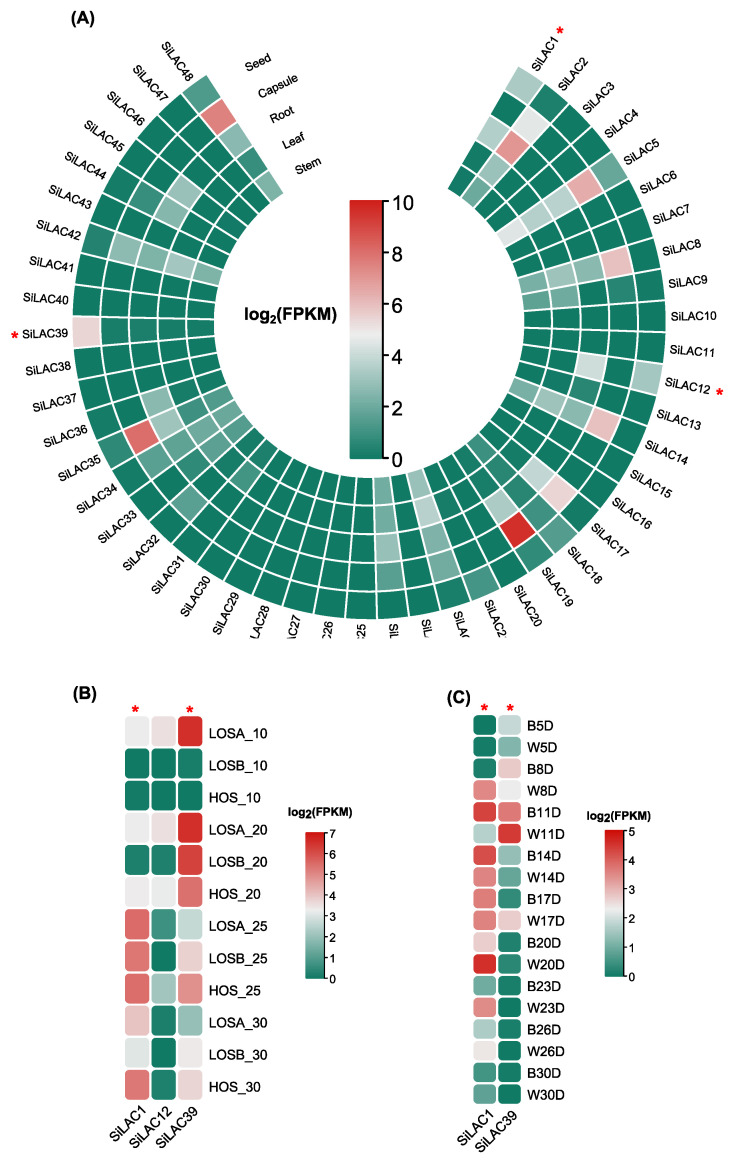
Expression profile of sesame laccase genes in different tissues: (**A**) seed, root, leaf, stem and capsule from Zhongzhi13; (**B**) Seed from ZZM4728 (HOS), ZZM3495 (LOSA), and ZZM2161 (LOSB); (**C**) Seed from black seed Zhongzhi No. 33 (B) and white seed Zhongzhi No.1 (W) varieties. Candidate genes for each set of tissues are highlighted with a red star symbol.

As for the laccase genes (Figure 7), *SiLAC1*, *SiLAC12*, and *SiLAC39* came out on the top in the first filtering round (Figure 7A). From this set, *SiLAC39* showed the peak of expression in both low and high oil content varieties at 20 days after anthesis. Similarly, *SiLAC1* expression was higher at 25 days after anthesis regardless of the type of variety (Figure 7B). Furthermore, the expression of the two later genes was checked in the black versus white varieties (Figure 7C). Interestingly, *SiLAC1* exhibited a higher expression in the white seed variety (Zhongzhi No.1) at the early stage of the seed development (8 days after anthesis) compared to the black one (Zhongzhi No.33). Similarly, *SiLAC39* expression was differentially higher in white seed variety (Zhongzhi No.1) at both 11 and 17 days after anthesis.

**Figure 8:**
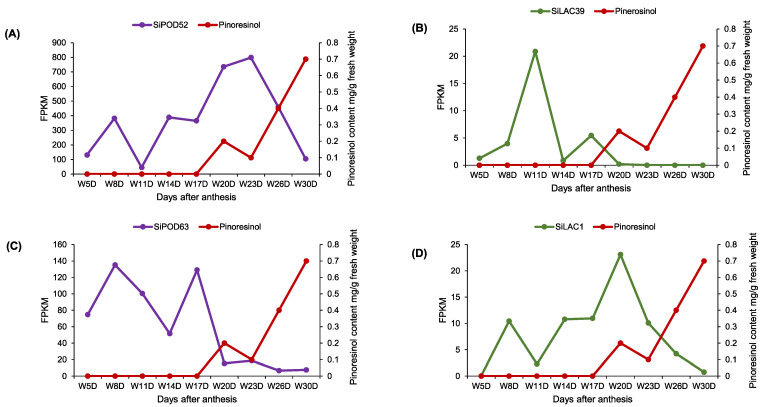
FPKM variation of candidate peroxidase and laccase genes following sesame seed development and pinoresinol content in white seed genotype Zhongfengzhi No1. Pinoresinol content values were obtained from Ono et al. [23] study. *SiPOD52* (**A**), *SiLAC39* (**B**), *SiPOD63* (**C**), and *SiLAC1* (**D**) variations following pinoresinol content were depicted during the seed development stages starting from five days after anthesis (W5D) to 30 days after anthesis (W30D). Pinoresinol content was colored in red. Peroxidase and laccase gene expression counts were colored in purple and green, respectively.

It is worth noting that all candidate genes fall into the principle according to which they should belong to the core gene repertoire of the sesame pan-genome and also, follow the pinoresinol synthesis kinetic, as described by Ono et al. [23] and depicted in Figure 8.

Briefly, the kinetic of the lignans biosynthesis suggests the expression of oxidative enzymes (potentially here peroxidase and/or laccase) at an upstream stage (from +(−)coniferyl alcohol to +(−)pinoresinol synthesis) of the lignans biosynthesis. Therefore, pinoresinol synthase is supposed to be expressed at an early stage of the seed development to enable downstream biosynthesis, resulting in +(−) sesamin, +(−) sesamolin, and +(−) sesaminol at the maturity stage (~30 days after anthesis). Thus, by combining the pinoresinol content extracted from sesame seed by Ono et al. [23] and the transcript data sets (Figure 8), we selected two peroxidase (*SiPOD52*, *SiPOD63*) and two laccase (*SiLAC1*, *SiLAC39*) genes. Globally, the FPKM values of the four candidate genes were declining at 23 days after anthesis (approximatively one week before the maturation stage) (Figure 8). The *SiPOD50* gene was filtered out since it was constantly expressed at all development stages of the seed (Figure 7C), which does not match the lignan biosynthesis kinetic. Therefore, in downstream bioinformatic analyses (cis-acting elements, orthologs identification and gene ontology analyses), only *SiPOD52*, *SiPOD63*, *SiLAC1*, and *SiLAC39* genes were selected.

### 3.3. Cis-Acting Elements, Related Transcription Factors, and Functional Attributes

**Figure 9:**
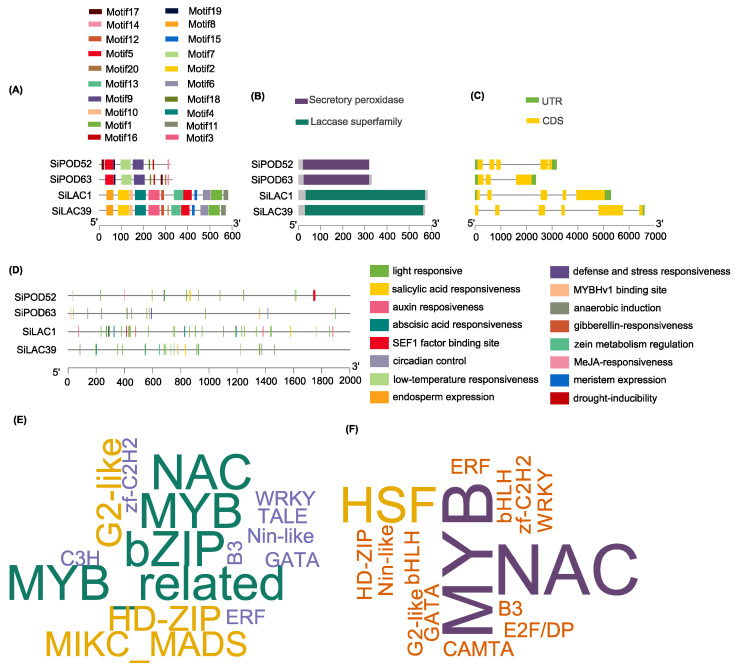
Bioinformatic analysis of candidate peroxidase and laccase genes. Protein motifs (**A**), domain (**B**) and gene structure (**C**), cis-acting elements functional attributes (**D**), and transcription factor enrichment word clouds for laccase (**E**) and peroxidase (**F**) candidate genes.

Although the candidate genes shared similar gene structures (Figure 9A–C), a wide diversity of regulation and functional characteristics were highlighted through the cis-element analysis (Figure 9D), including hormonal response, light response, abiotic stress response, and physiological development. This suggests that *SiPOD* and *SiLAC* genes might be involved in a broad spectrum of biological processes in sesame.

Knowing the important role of transcription factors in gene regulation, we performed a transcription factor-oriented enrichment analysis by using all *SiPOD* and *SiLAC* genes to identify candidate transcription factors (TF) potentially involved in peroxidase and laccase gene regulation.

A panel of TF families was predicted, among which v-myb avian myeloblastosis viral oncogene homolog (*MYB*), *NAM* (no apical meristem), *ATAF1-2* (Arabidopsis thaliana activating factor), CUC2 (cup-shaped cotyledon) (*NAC*), Basic leucine zipper (*bZIP*), Heat shock factors (*HSF*), Homeodomain-leucine zipper (*HD-ZIP*), MIKC-type MADS-box (*MIKC_MADS*) were the most abundant. As depicted in Figure 9E,F, MYB is the most predominant TFs indicating their putative regulatory role in the expression of both peroxidases and laccases. Using transgenic *A. thaliana* lines, Shen et al. [96] showed that the sweet cherry (*Prunus avium* cv. Hong Deng) *R2R3 MYB* was able to alleviate salt stress and provide anti-bacterial resistance through the activation of peroxidases and accumulation of anthocyanin. Furthermore, co-expression of *A. thaliana* laccases (*lac4* or *lac17*) with *MYB63* genes is known to rescue dwarfism in *A. thaliana* mutant lines [97].

Looking at the orthologs of the candidate genes in other taxa by a phylogenetic approach with SHOOT [72], we found homologous sequences in oil- and non-oil crops including *Solanum lycopersicum*, *Arabidopsis thaliana*, *Brassica oleracea*, *Gossypium raimondii*, *Glycine max*, *Triticum aestivum*, *Oryza sativa*, and *Zea mays* (Table S5).

The GO annotation supported the hypothesis that the all-candidate peroxidase genes are involved in the hydrogen peroxidase catabolic process (GO:0042744) with a heme-binding (GO:0020037) and peroxidase activity (GO:0004601) as main molecular functions. Regarding laccase, the GO annotation indicated that *SiLAC1* and *SiLAC39* may be related to the degradation of lignin (GO:0046274), with copper ion binding (GO:0005507) and oxidase activity (GO:0016491) as major molecular functions. From the GO annotation results, both *SiPOD* and *SiLAC* genes were predicted to have an oxidative role, which is a key requirement for the transformation of (+)- coniferyl alcohol into (+)- pinoresinol.

To assess the validity of the RNA-seq data, a qRT-PCR experiment was performed using the selected genes (Figure S7A). The trends of changes in the relative expression were highly consistent with those of the transcriptome sequencing data (R^2^ = 0.8505) (Figure S7B); indicating that the RNA-seq data were reliable.

As matter of fact, peroxidase and laccase genes were reported to be able to oxidize coniferyl and p-coumaryl alcohols, acting as catalysts during cell wall lignification in *Zinnia elegans* [98,99,100,101,102]. Therefore, the suggested genes are a valuable candidate for functional validation and ultimately, for usage in the pharmaceutical and food industries through bioengineering. Since sesame is recalcitrant to genetic transformation using classical methods [2], the hairy roots method might be a valuable alternative path for in vitro production of sesame lignans as demonstrated by Ogasawara et al. [103].

## 4. Conclusions

Candidate genes for peroxidase (83 genes) and laccase (48 genes) were identified in the genome of sesame. Gene count for both genes varied considerably between varieties. Taking advantage of a large panel of transcriptome data and stringent filtering, four genes (*SiPOD52*, *SiPOD63*, *SiLAC1*, and *SiLAC39*) were proposed as candidates for encoding (+)-pinoresinol synthase. The genes were predicted to interact with a wide range of transcription factors, indicating that they are involved in diverse physiological processes. The findings of this study open a way for functional investigation of the candidate genes and eventually for the genetic improvement of sesame regarding the synthesis of lignans.

## Data Availability

The peroxidase and laccase genes presented in this study are available in Tables S2 and S3. The NCBI SRA accessions are listed in Table S1. The sesame reference genome assembly and annotation of Zhongzhi13 are available under the NCBI project number PRJNA186669.

## Supplementary Materials

The following supporting information can be downloaded at: https://www.mdpi.com/article/10.3390/life12081200/s1, Figure S1: Circos plot showing paralogous peroxidase genes exhibiting segmental duplication in sesame; Figure S2: Circos plot showing paralogous laccase genes belonging to segmental duplication pattern; Figure S3: Circos plot showing synthenic peroxidase genes between *Sesamum indicum* and *Arabidopsis thaliana*; Figure S4: Circos plot showing synthenic laccase genes between *Sesamum indicum* and *Arabidopsis thaliana*; Figure S5: Motif structure of sesame peroxidase genes; Figure S6: Motif structure of sesame laccase genes; Figure S7: qRT-PCR results of the target genes and correlation with RNA-seq data; Table S1: Information relative to the SRA accessions of RNA sequencing data used in the present study; Table S2: List of primers used in the quantitative real time-PCR analysis; Table S3: List of identified peroxidase genes in *Sesamum indicum* var. Zhongzhi13; Table S4. List of identified laccase genes in *Sesamum indicum* var. Zhongzhi13, Table S5. List of candidate genes, functional attributes from TAIR database and their orthologous sequences.
