# Supplementary material for: Characterization of Peroxidase and Laccase Gene Families and In Silico Identification of Potential Genes Involved in Upstream Steps of Lignan Formation in Sesame
Source: Life (Basel). 2022 Aug 8;12(8):1200. doi: 10.3390/life12081200 (PMC9410177; doi:10.3390/life12081200)
Supplement: Supplementary file 1 [file life-12-01200-s001.zip › Supplementary_Figures.pdf]

Supplementary Figures

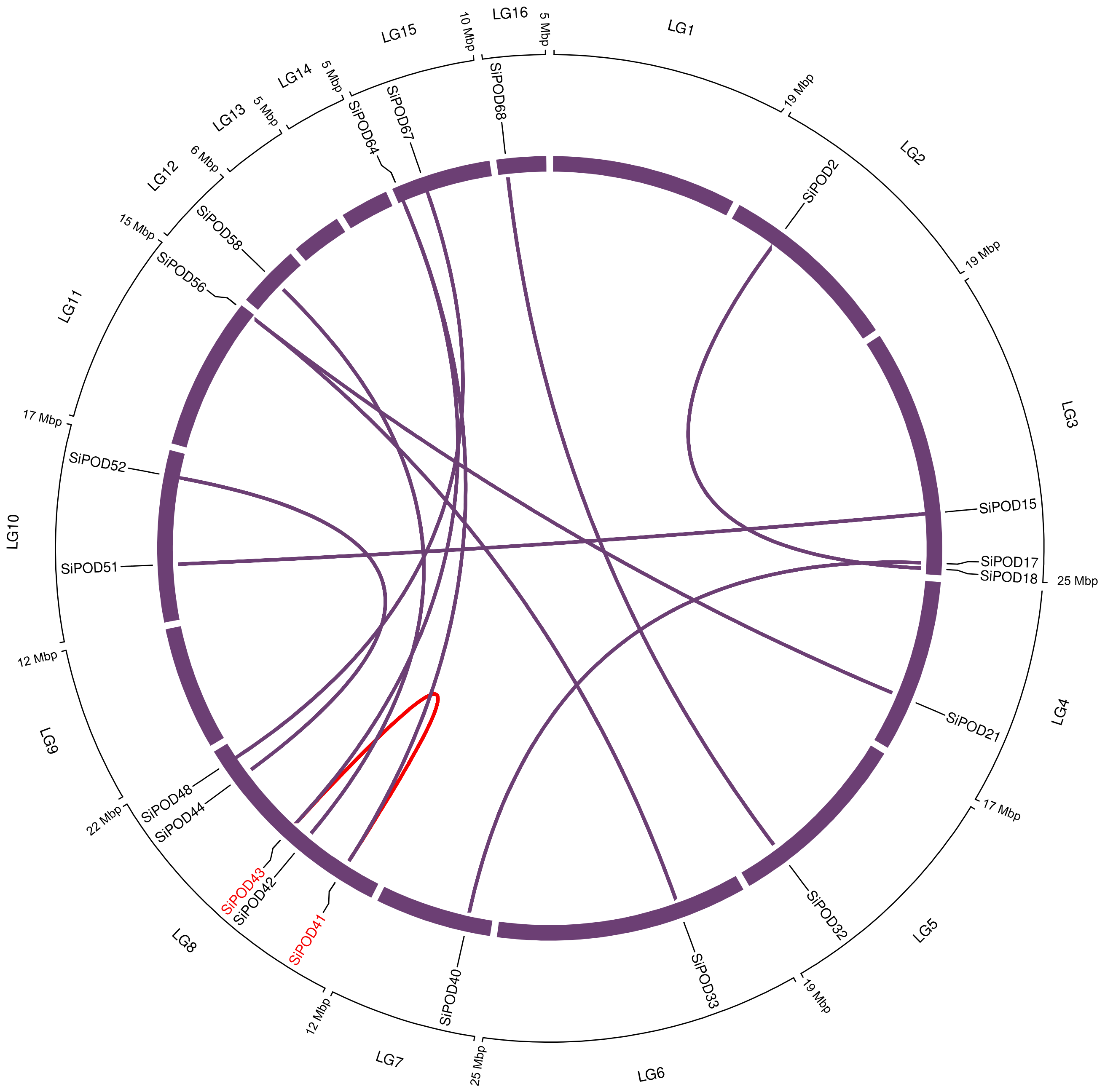

Figure S1. Circos plot showing paralogous peroxidase genes exhibiting segmental duplication in sesame. Genes colored in red are tandem duplicated.

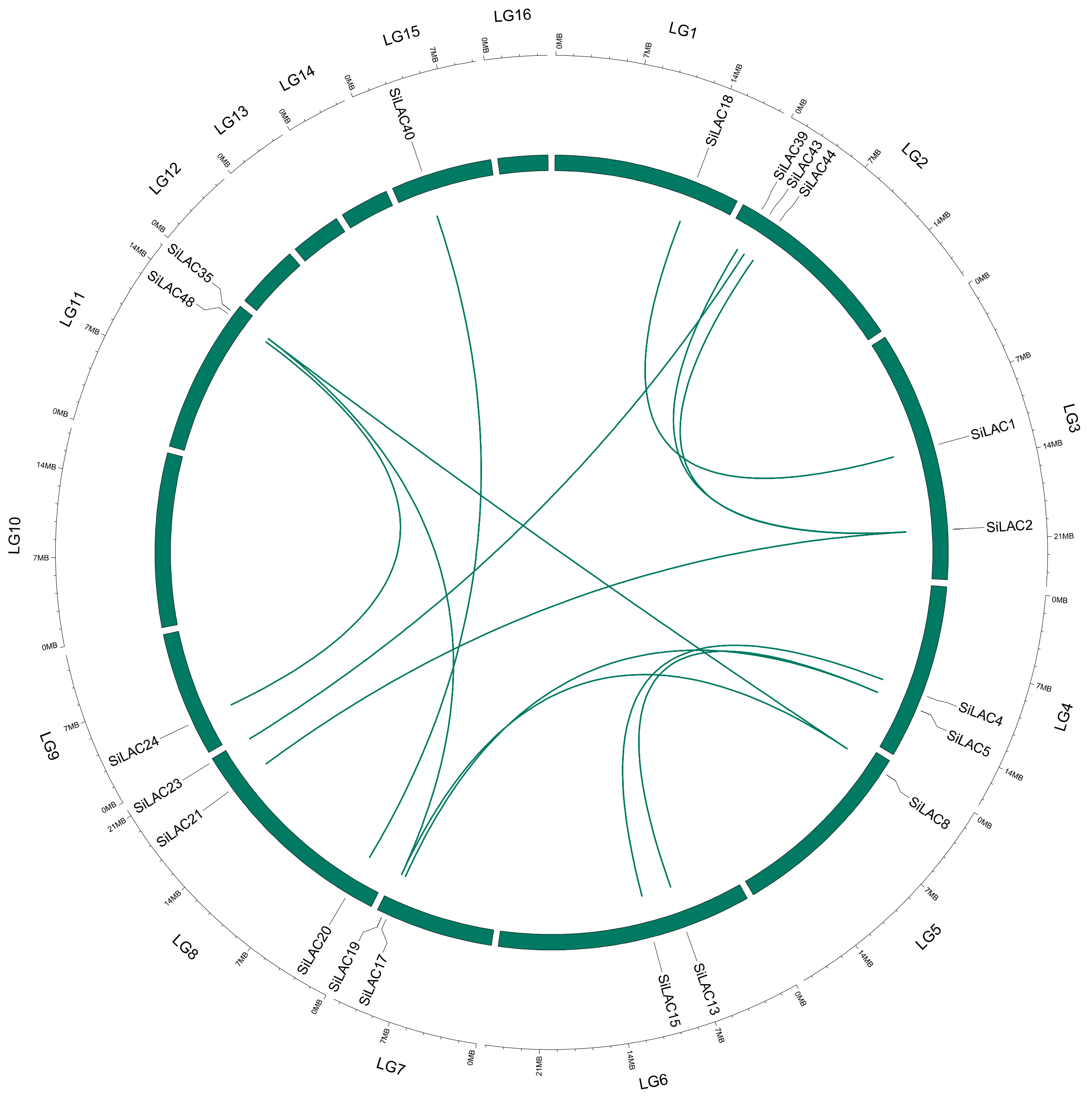

Figure S2. Circos plot showing paralogous laccase genes belonging to segmental duplication pattern.

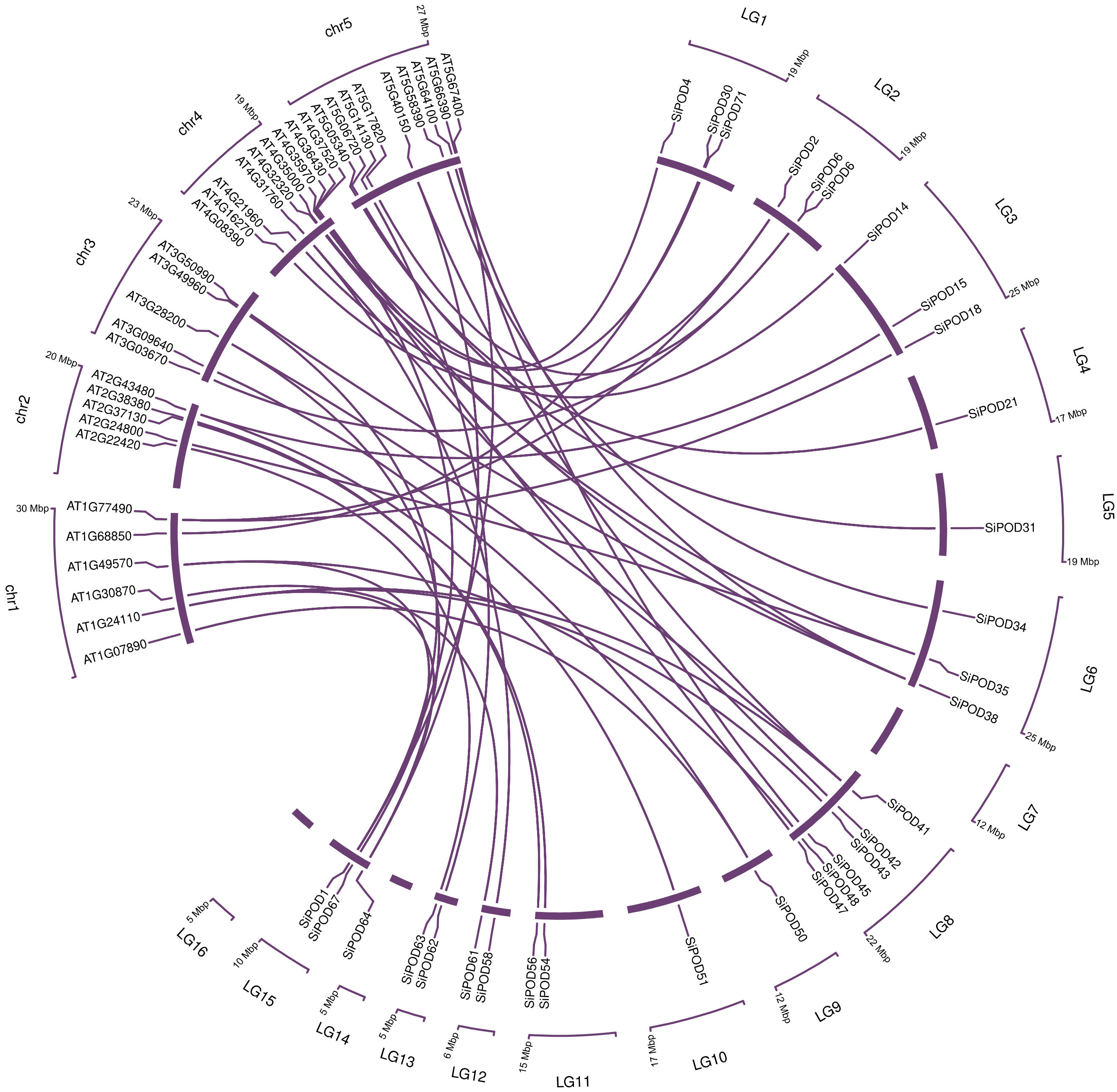

Figure S3. Circos plot showing synthenic peroxidase genes between *Sesamum indicum* and *Arabidopsis thaliana*.

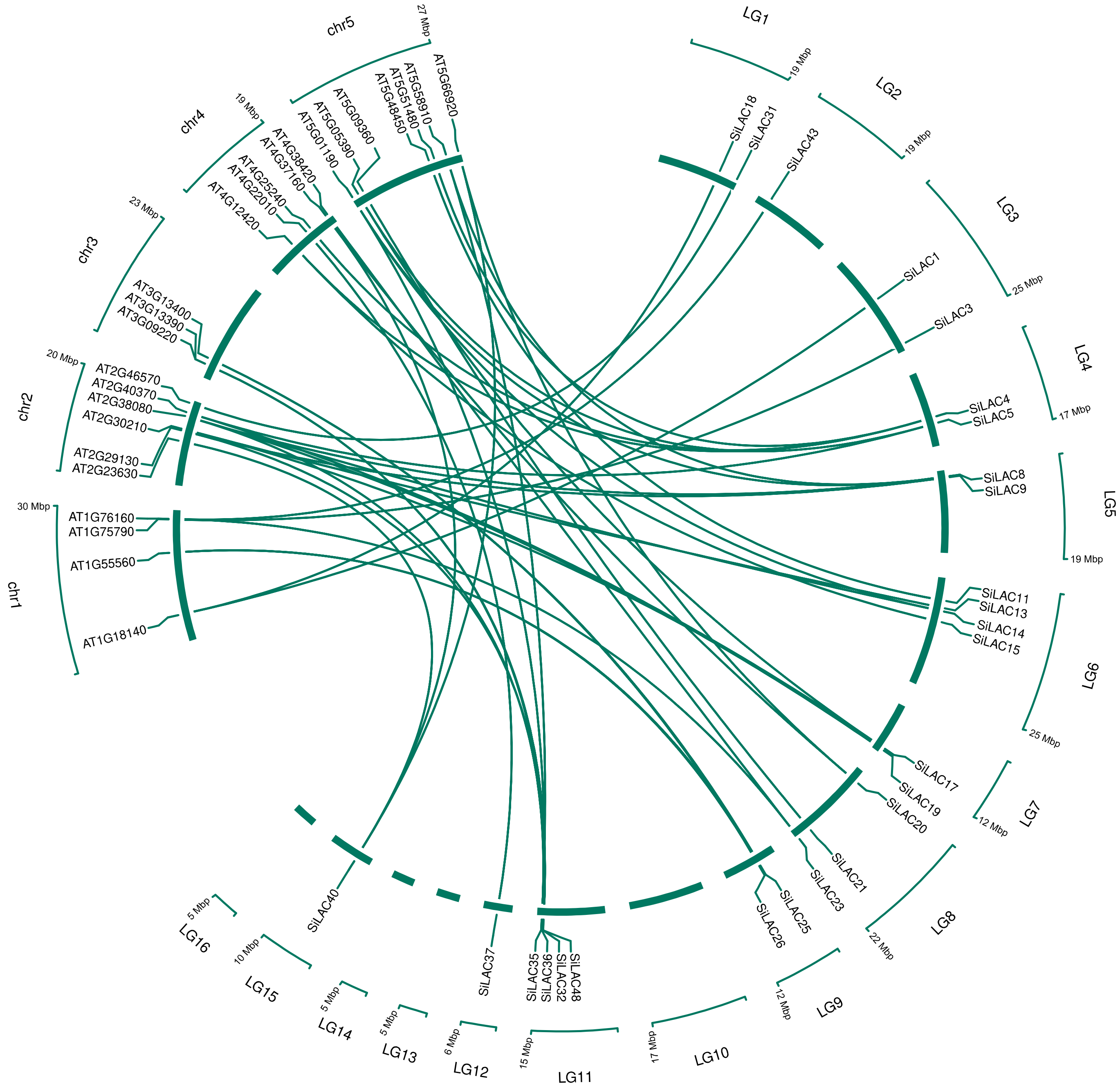

Figure S4. Circos plot showing synthenic laccase genes between *Sesamum indicum* and *Arabidopsis thaliana*.

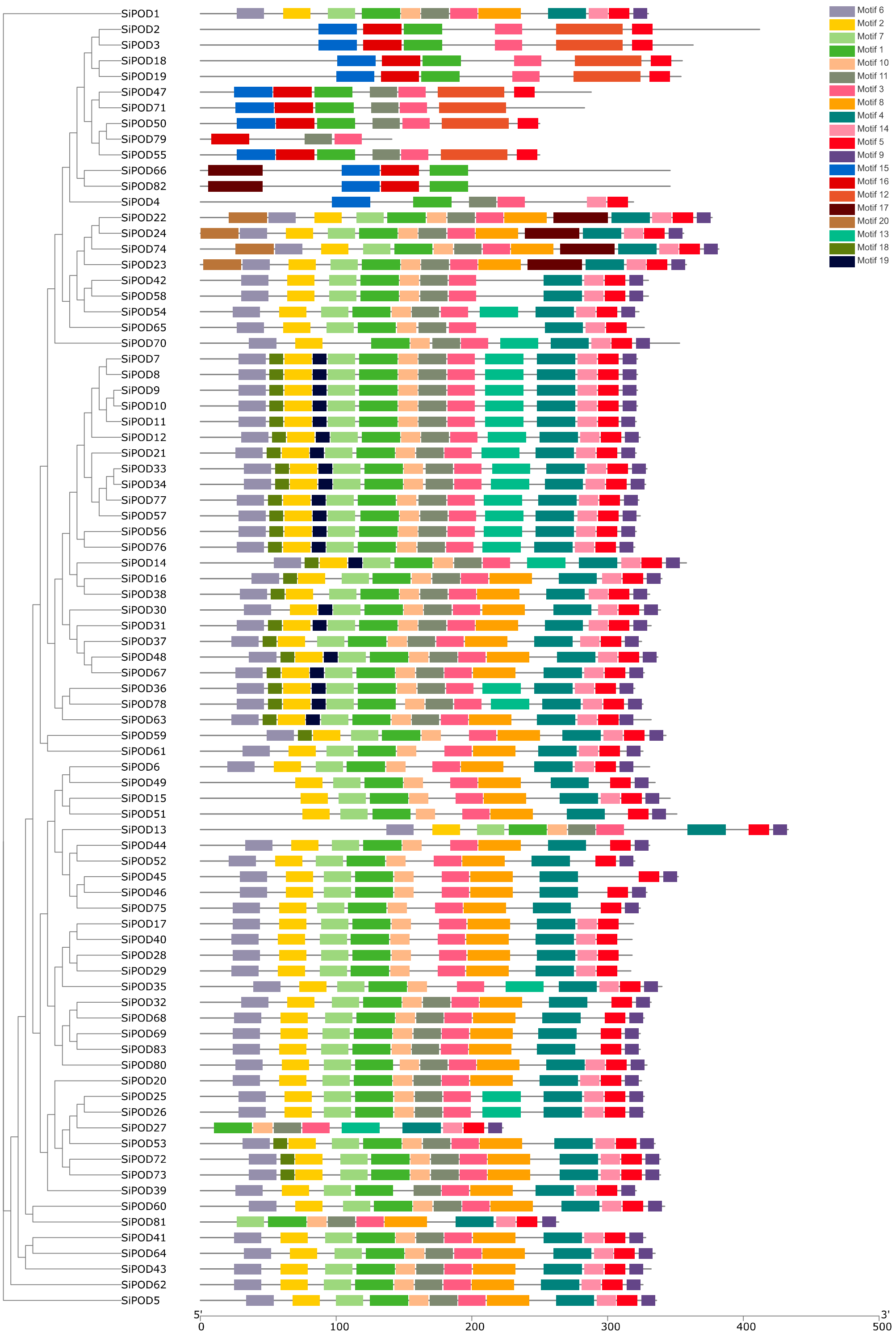

Figure S5. Motif structure of sesame peroxidase genes

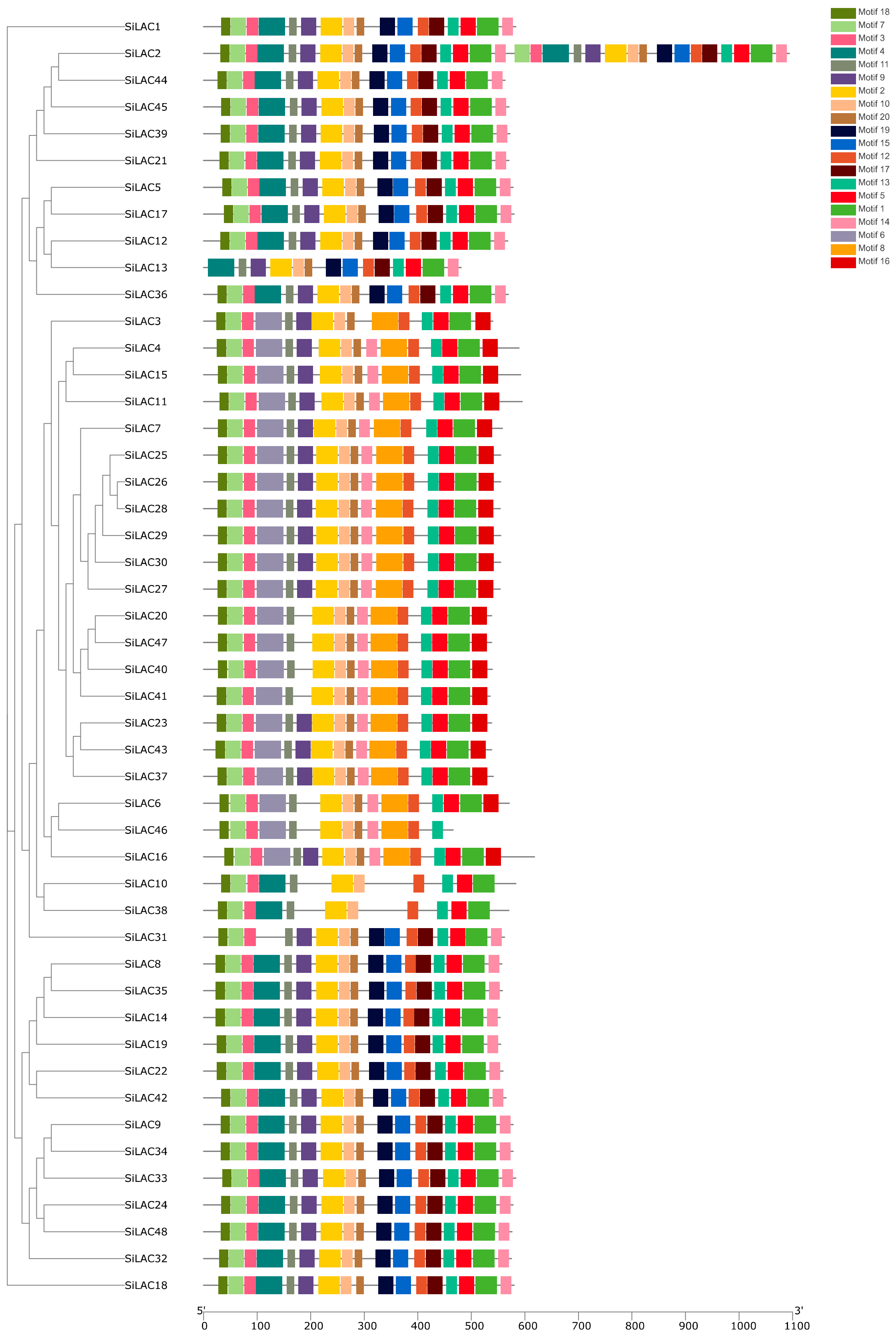

Figure S6. Motif structure of sesame laccase genes

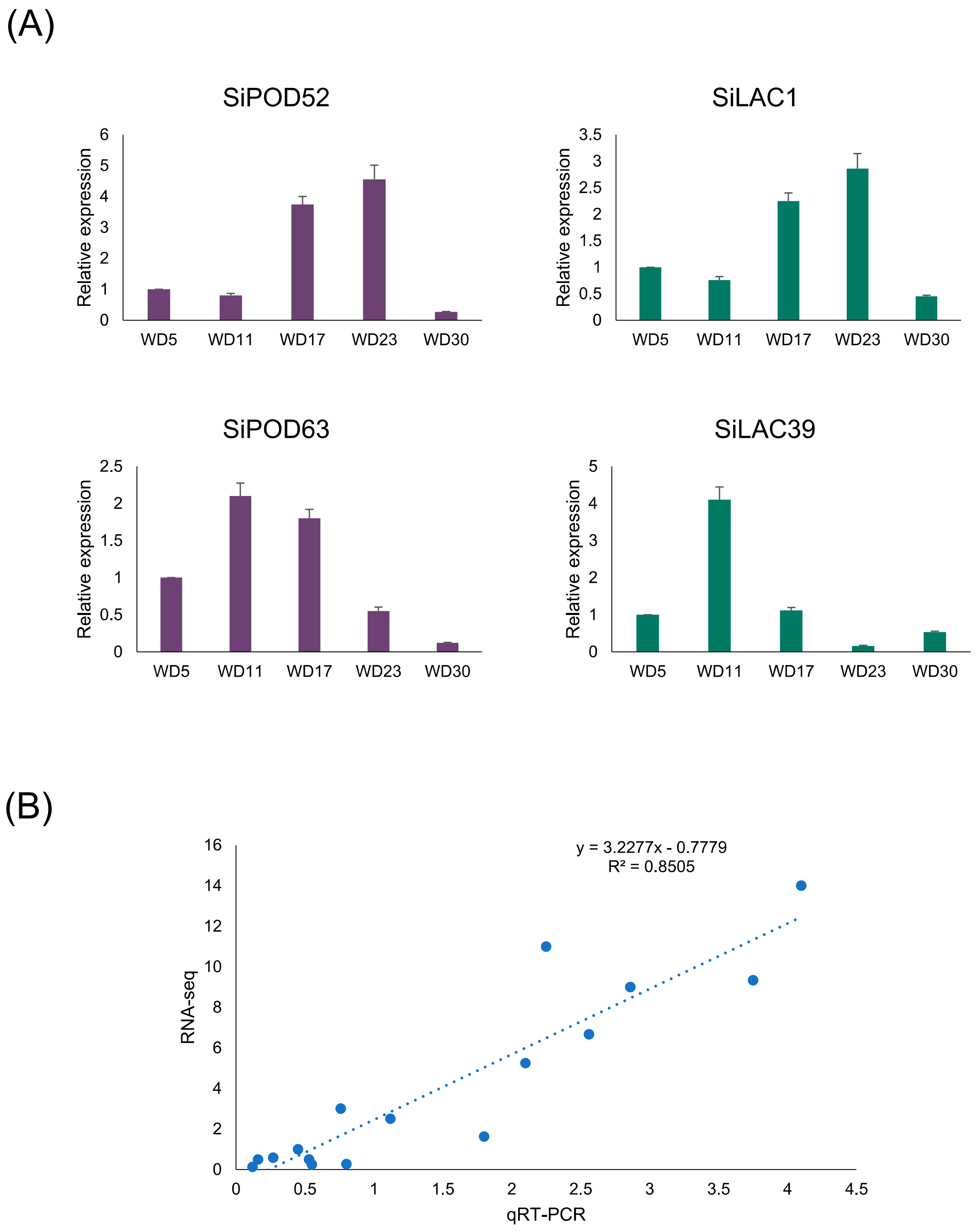

Figure S7. qRT-PCR results of the target genes (A) and correlation (B) with RNA-seq data
